# Identification of the potential novel biomarkers as susceptibility gene for Wilms tumor

**DOI:** 10.1186/s12885-021-08034-w

**Published:** 2021-03-25

**Authors:** Li Liu, Zhe Song, Xu-Dong Gao, Xian Chen, Xiao-Bin Wu, Mi Wang, Yu-De Hong

**Affiliations:** 1 Department of Urology, The Second Hospital, University of South China, Hengyang, 421001 Hunan China; 2 College of Health Science and Nursing, Wuhan Polytechnic University, Wuhan, 420000 China

**Keywords:** Wilms tumor, Biomarkers, Recurrence, Overall survival

## Abstract

**Background:**

Wilms tumor (WT) is the most common malignant renal tumor in children. The aim of this study was to identify potential susceptibility gene of WT for better prognosis.

**Methods:**

Weighted gene coexpression network analysis is used for the detection of clinically important biomarkers associated with WT.

**Results:**

In the study, 59 tissue samples from National Cancer Institute were pretreated for constructing gene co-expression network, while 224 samples also downloaded from National Cancer Institute were used for hub gene validation and module preservation analysis. Three modules were found to be highly correlated with WT, and 44 top hub genes were identified in these key modules eventually. In addition, both the module preservation analysis and gene validation showed ideal results based on other dataset with 224 samples. Meanwhile, Functional enrichment analysis showed that genes in module were enriched to sister chromatid cohesion, cell cycle, oocyte meiosis.

**Conclusion:**

In summary, we established a gene co-expression network to identify 44 hub genes are closely to recurrence and staging of WT, and 6 of these hub genes was closely related to the poor prognosis of patients. Our findings revealed that those hub genes may be used as potential susceptibility gene for clinical diagnosis and prognosis of this tumor.

**Supplementary Information:**

The online version contains supplementary material available at 10.1186/s12885-021-08034-w.

## Background

Wilms tumor (WT) is common abdominal malignancy in children and accounts for up to 95% of renal tumors in children [1]. This tumor is an embryonal childhood tumor of metanephric origin, as it is histologically similar to the early stages of nephrogenesis, and many of the genetic changes that support the disease occur in genes associated with fetal kidney genes [2]. With the development of modern multimodality therapy, favorable histology WT survival has been achieved in more than 90% [3–5]. Of course, the described above does not include patients with advanced disease, bilateral WT patients and patients with recurrence. For approximately 10% of patients with high-risk subtypes of WT, the treatment outcome is not optimistic. Meanwhile, a challenge for all WT patients is recurrence. Approximately 15% of favorable histology WT patients will experience recurrence, and the overall survival of patients with recurrence can drop to 40 to 80% [6, 7]. However, although short-term survival is high in patients with WT, long-term survival is reduced in patients with WT due to adverse therapeutic effects of cancer treatment, such as renal insufficiency, secondary malignancies, and heart failure. In addition to technical improvements, it is also important to clarify the stage of WT and avoid overtreatment. And, the high recurrence rate of WT with favorable histology indicates that new therapies are needed to improve the prognosis. Therefore, it’s urgent to detect potential susceptibility gene associated with WT. The samples data for this study came from the National Cancer Institute (NCI) with large, global database of cancer-related genetic variations. Samples were constructed co-expression network according to weighted gene co-expression network analysis (WGCNA), which was performed using the WGCNA package for R [8]. WGCNA, which is a systems biology method for the analysis of microarray data, is widely used for the detection of potential biomarkers. The application of WGCNA is based on a scale-free network distribution, and its advantage over other research methods may be that it can maximize the use of effective data information. When transforming the correlation matrix into the adjacency matrix, we choose β to weight the correlation coefficient, “polarize” the correlation coefficient and make the correlation and noncorrelation more significant. WGCNA is widely used as a powerful data-driven tool to study the expression of cancer genes and to help understand the development mechanisms of various cancers, such as clear cell renal cell carcinoma (ccRCC), hepatocellular carcinoma (HCC), lung cancer.

In the study, a weighted gene co-expression network was performed to identify modules that are significantly related to WT. Then the top hub genes are screened out in the module. These genes might as potential susceptibility gene to reduce recurrence for patients with WT, avoid overtreatment by scientific pathological staging, and thus minimize toxicity treatment with other conditions unchanged, leading to better prognosis and better long-term survival. This study may have important reference value for potential susceptibility gene of this disease.

## Method

### Data collection and data preprocessing

Raw gene expression profiles were downloaded from NCI (https://ocg.cancer.gov/programs/target/data-matrix). All tissue samples were from the platform named GPL96 [HG-U133A] Affymetrix Human Genome U133A Array. After the raw expression data were corrected by the robust multi-array averaging (RMA) algorithm [9], the nsFilter algorithm was used to filter the data for the next analysis. There were 6201 probes and 59 primary tumor tissue samples through filtering. The samples were named by the provider that indicated gender, age at diagnosis, overall survival time, event (recurrent) free survival time and disease stage.

### Weighted genes co-expression network analysis

WGCNA was carried out to screen for significantly stable modules that are related to WT. The key to constructing the gene co-expression network by the WGCNA method [10] is “weighted”; specifically, the selection of soft threshold β is the key link for subsequent analysis. By using the scale-free topological criterion to select a soft threshold [11], the co-expression network has a high biological signal and is closer to the scale-free network distribution. Generally, when R^2^ > 0.8, the network was considered approximately to the scale-free network distribution. In short, the correlation matrix was converted into the adjacency matrix based on the soft threshold β, and the specific calculation is a_ij=_|cor (x_i_, x_j_)|^β^, X_i_ and x_j_ are the nodes i and j of the network. Then, the adjacency matrix was transformed into a topological overlap matrix (TOM) after a series of complex calculations. TOM provides a simplified diagram of the network, allowing the visualization of the network and facilitating the identification of network modules. Then the TOM graph is analyzed by average linkage hierarchical clustering based on the phase dissimilarity (1-TOM). Modules, clusters of highly interconnected genes in co-expression network, were identified after performing cluster analysis and dynamic tree shearing. The height cut-off value of dynamic tree shearing was guided by TOM. In general, the number of genes in each module is 30 and above. Meanwhile, each module was marked with a different color, and all non-characteristic genes were assigned to gray module, and the grey module was not involved in subsequent analysis [11].

### Module preservation analysis and identification of clinically significant modules

Module preservation analysis was carried out to measure the stability of each module defined. The stability of the module is measured by the Z summary score (Z-score) and medianRank [12]. The higher the Z-score is, the better the module is preserved, and the more reliable the subsequent analysis will be. In general, if the Z-score is greater than 10, it is considered that the module is well-preserved. Moreover, the medianRank of the modules close to zero indicates the high degree of module preservation.

The gene modules most relevant to clinical features were selected for subsequent analysis. In addition to qualitative analysis, quantitative calculations involving modules eigengene (ME), gene significance (GS) and module significance (MS) were performed. ME is the first principal component in the gene module and may represent the gene expression profile of the whole module. GS can be understood as the correlation between genes and traits. The higher the GS of a given gene is, the more closely the gene is related to clinical characteristics, and the more significant the biological significance is [8]. MS represents the average value of GS in the module.

### Identification and validation of hub genes and functional annotation

Select hub genes in two ways: those that have been shown in previous studies to be strongly associated with disease and those that are highly connected in the gene co-expression network [13]. The study focused on the latter approach. Hub genes or key genes are those that are significantly related to clinical characteristics and are highly connected with other genes in a gene module [8]. The former can be measured by geneTraitSignificance (geneTraitSignificance> 0.20), and the latter can be measured by geneModuleMembership (geneModuleMembership> 0.80). In addition, protein-protein interaction (PPI) network was visualized by Cytoscape (http://www.cytoscape.org/) package. Proteins that are highly connected in the PPI network are more important than those that are not, and the same is true for the corresponding genes [14]. After the co-expression network, the analysis of PPI network is equivalent to the scoring of genes from the perspective of proteins, which is conducive to the further identification and verification of hub genes. In PPI networks, we associate the first two values to identify some more biologically significant genes as hub genes for subsequent analysis. At the same time, to better understand the mechanism of gene action and to facilitate subsequent analysis, pathway enrichment analysis of hub genes in selected modules needed to be conducted. We uploaded genes to Enrichr for enrichment analysis and functional annotation [15].

Furthermore, 224 samples from NCI to test whether the hub genes were significantly expressed in the sample based on One-way analysis of variance (one-way ANOVA) To verify the hub genes, specifically, is to verify the differential expression of the hub gene in other independent data sets is statistically significant.

### Hub gene evaluation

To assess the relationship between hub genes and WT patients, the “survival” package of R 3.5.2 software was used for the log-rank test and Kaplan-Meier survival analysis. The Kaplan-Meier method is a nonparametric survival analysis method, which is widely used in survival analysis of cancer research as a smart statistical treatment method of survival time [16]. In the section, patients are usually divided into two groups according to each gene expression (high vs. low). The corresponding Kaplan-Meier estimation value was calculated, and the Kaplan-Meier survival curve was determined. Furthermore, log-rank was used to test whether the difference in survival time between the two groups was statistically significant. Furthermore, time-dependent receiver operating characteristic (ROC) analysis was used to evaluate diagnosis value of hub genes.

## Results

### Weighted co-expression network construction

**Fig. 1:**
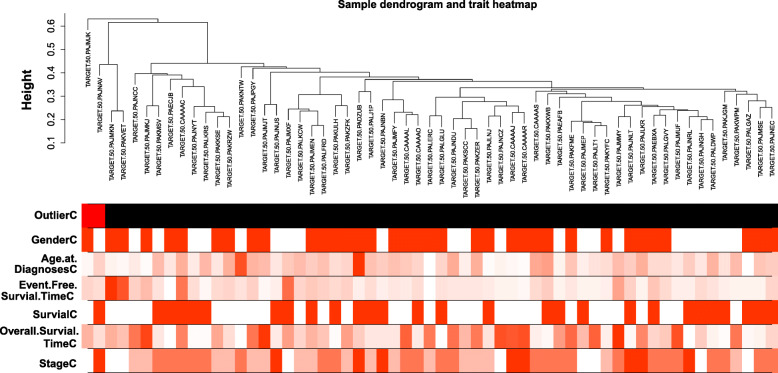
Clustering dendrogram of 59 tumor samples and the clinical traits. Note: The color intensity was proportional to gender, age at diagnoses, event free survial time, survial, sverall survial time and stage

After a series of data preprocessing including filtering, 6201 probes and 59 samples were obtained. When the correlation matrix was transformed into an adjacency matrix, as shown in Additional file 1, β = 11 (scale free R^2^ = 0.88) was selected as the weighted coefficient value. The scale free is closest and higher than 0.85 for the first time. Two samples with Z. K value <− 2.5 (TARGET.50. PAJMJK and TARGET.50. PAJNAV) were filtered out (Fig. 1). Finally, 13 modules were identified (Additional file 2).

### Module preservation analysis and identification of clinically significant modules

**Fig. 2:**
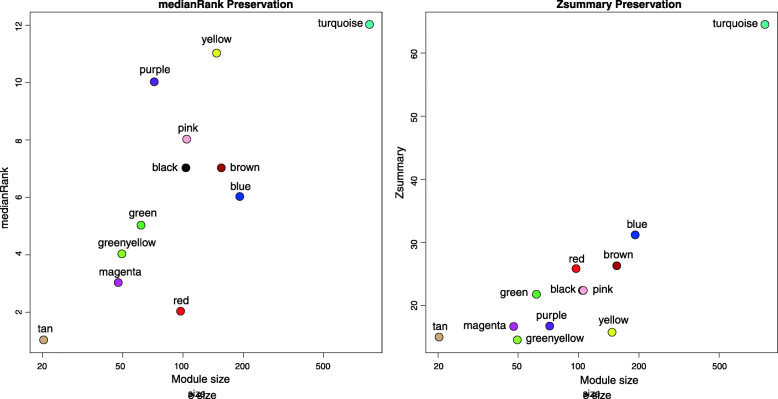
The medianRank and Zsummary statistics of the module preservation. Note: The medianRank of the modules close to zero indicates the high degree of module preservation, and the Zsummary of the modules close to zero indicates the low degree of module preservation

The study conducted module preservation analysis use “modulePreservation” and determined whether the modules are preserved according to two main parameters: the Z-score and medianRank. As shown in Fig. 2, the Z-scores of the green, pink, red, brown, black, blue module and turquoise module are all above 20, and the turquoise module is highest. The high Z-scores of these modules indicate that these modules are well preserved, but since Z-scores are highly dependent on the size of modules, we also need to analyze the medianRank. Although the Z-score of turquoise module indicates that it is well preserved, the medianRank of turquoise module is not optimistic, indicating that the module may be unstable. However, the two values of green, pink and blue modules indicate that these modules have good stability.

**Fig. 3:**
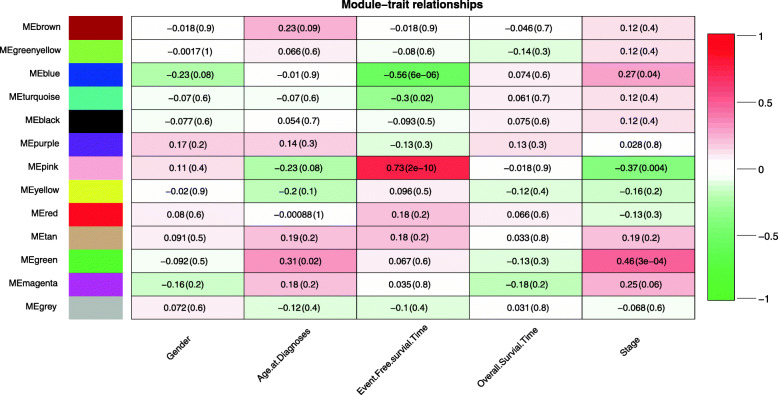
Module-trait relationships. Note: Heatmap of the correlation between module eigengenes and clinical traits of WT. Each module based on pattern of their co-expression

Three modules were prominent in the module-trait relationship (Fig. 3): the green module with (r = 0.46, *P* = 3e-04) highly correlated with the disease stage, the blue module (r = − 0.56, *P* = 6e-06) and pink module (r = 0.73, *P* = 2e-10) with highly correlated with event-free survival time. But beyond all that, green modules (cor = 0.65, *P* = 1.7e-19, Additional file 3), blue modules (cor = 0.5, P = 3e-15, Additional file 4) and pink modules (cor = 0.62, *P* = 6.5e-13, Additional file 5) were displayed with high genetic significance and module membership.

### Identification and validation of hub genes and functional annotation

**Fig. 4:**
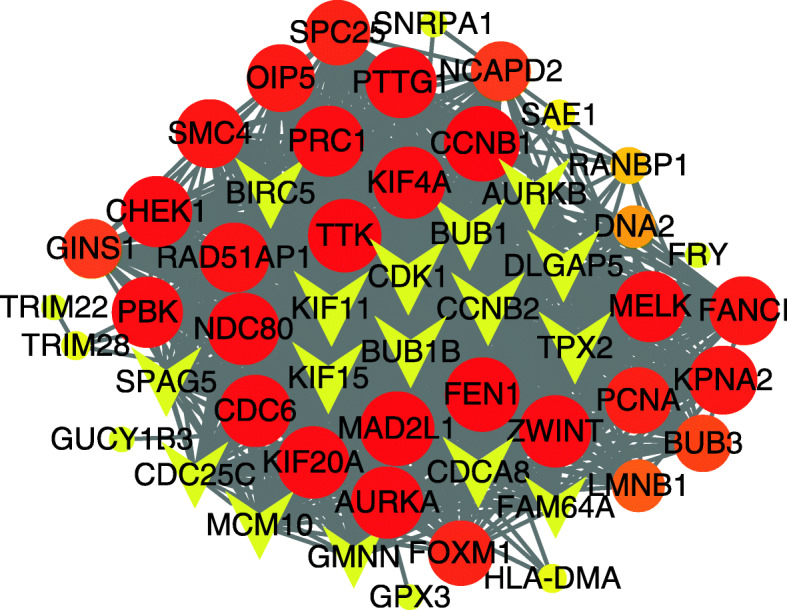
The protein-protein network of the candidate genes in blue module and pink module. Note: Each graph represents a gene, and the size of the graph is proportional to the degree of connectivity, and the higher the degree of connectivity between the orange and red genes. The arrow represents the candidate genes in the network

**Fig. 5:**
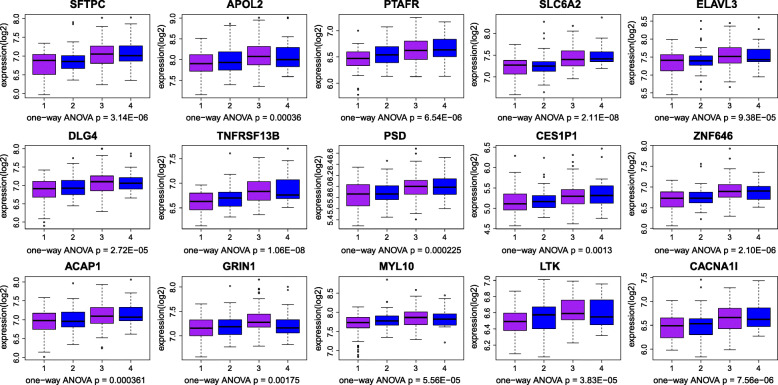
Boxplot for identification of hub genes in the green module

**Fig. 6:**
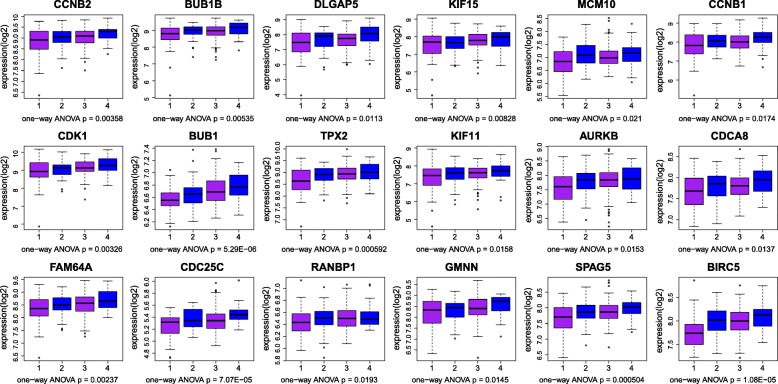
Boxplot for identification of hub genes in the blue module

**Fig. 7:**
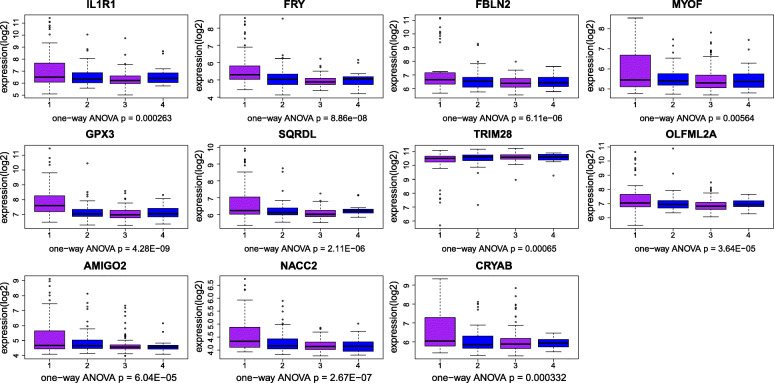
Boxplot for identification of hub genes in the pink module

The genes with geneTraitSignificance> 0.20 and geneModulesMemership> 0.80 were defined as candidate genes, and the hub gene was not only the candidate gene but also the gene ranked in the top 10% of the module. Candidate genes in blue and pink modules were constructed a PPI network (Fig. 4) for they associated with the same clinical characteristics. Due to the superior PPI network connectivity of the blue module gene, the PPI network was also used in the selection of the hub genes in the blue module gene. Therefore, the study selected 15 genes from the green module, 18 genes from the blue module, and 11 genes from the pink module as the hub genes in Table 1. Finally, one-way ANOVA was used to verify the hub genes that finally defined. The verification results of the green module, blue module and pink module are shown in Figs. 5, 6 and 7, respectively. The differential expressions of 44 genes were statistically significant (*P* < 0.05).

**Table 1:** Hub genes from the green module, blue module, and pink module

ProbeID	Gene Symbol	ENTREZ_GENE_ID	GeneModuleMembership	GeneTraitSignificance
**Green Module**				
215454_x_at	SFTPC	6440	0.9655947	0.4884202
221013_s_at	APOL2	23,780	0.945633	0.4684544
206278_at	PTAFR	5724	0.9403485	0.4480241
216611_s_at	SLC6A2	6530	0.9302162	0.4672082
206338_at	ELAVL3	1995	0.928487	0.4336345
210684_s_at	DLG4	1742	0.924651	0.4306517
207641_at	TNFRSF13B	23,495	0.9209938	0.4174775
208102_s_at	PSD	5662	0.9137638	0.4527653
206824_at	CES1P1	51,716	0.9117621	0.4020609
204876_at	ZNF646	9726	0.9109073	0.4119248
205212_s_at	ACAP1	9744	0.9108941	0.4238431
210782_x_at	GRIN1	2902	0.9107197	0.3644859
221660_at	MYL10	93,408	0.910379	0.4163593
207106_s_at	LTK	4058	0.9083481	0.4073561
208299_at	CACNA1I	8911	0.9071691	0.4603597
**Blue Module**				
202705_at	CCNB2	9133	0.904211166	0.502529831
203755_at	BUB1B	701	0.903761976	0.495338274
203764_at	DLGAP5	9787	0.896766419	0.520656849
219306_at	KIF15	56,992	0.88904352	0.484441899
220651_s_at	MCM10	55,388	0.882505533	0.420275022
214710_s_at	CCNB1	891	0.881525705	0.431836838
209642_at	BUB1	699	0.879762805	0.477439744
210052_s_at	TPX2	22,974	0.879693077	0.429308935
204444_at	KIF11	3832	0.876414663	0.562528262
209464_at	AURKB	9212	0.874473304	0.502557848
221591_s_at	FAM64A	54,478	0.865953299	0.505103365
202483_s_at	RANBP1	5902	0.86173565	0.474844941
218350_s_at	GMNN	51,053	0.861309011	0.482432439
203145_at	SPAG5	10,615	0.859158231	0.548259451
202095_s_at	BIRC5	332	0.854527142	0.405922833
221520_s_at	CDCA8	55,143	0.826403939	0.394343433
203214_x_at	CDK1	983	0.824308643	0.344276904
205167_s_at	CDC25C	995	0.808726515	0.519843377
**Pink Module**				
202948_at	IL1R1	3554	0.92457227	0.668226501
204072_s_at	FRY	10,129	0.900906947	0.620170176
203886_s_at	FBLN2	2199	0.885002611	0.704071608
201798_s_at	MYOF	26,509	0.875628221	0.541947295
201348_at	GPX3	2878	0.869676376	0.693172897
217995_at	SQRDL	58,472	0.869355878	0.66088755
200990_at	TRIM28	10,155	0.866953096	0.75816668
213075_at	OLFML2A	169,611	0.855803663	0.654017954
222108_at	AMIGO2	347,902	0.853035178	0.659563469
212993_at	NACC2	138,151	0.850368287	0.571281438
209283_at	CRYAB	1410	0.846869574	0.668086039

The candidate genes in each module were uploaded into the Enrichr database for Gene Ontology (GO) analysis and Kyoto Encyclopedia of Genes and Genomes (KEGG) pathway enrichment analysis. The candidate genes in the blue and pink modules were functionally annotated and enriched together. The GO functional annotation indicated that the blue and pink modules were enriched in sister chromatid cohesion, condensed chromosome kinetochore and spindle midzone, etc. (Table 2), and KEGG enrichment analysis showed that the blue and pink modules were enriched in the cell cycle, oocyte meiosis, etc. (Table 3**)**. GO and KEGG indicated that the expression mechanism of the green module was related to the excitatory postsynaptic potential, excitatory synapse (Table 4) and the ras signaling pathway (Table 5).

**Table 2:** GO functional annotation for genes in pink and blue modules

Series	Name	***P***-value	Adjusted p-value	Z-score	Combined score	Genes
GO Celluar Component	spindle midzone (GO:0051233)	2.19E-14	3.41E-12	−2.19	68.75	TPX2;PRC1;BUB1B;CDCA8;TTK;CDC6;KIF20A;KIF11;AURKB;AURKA
GO Celluar Component	condensed chromosome kinetochore (GO:0000777)	3.43E-14	3.41E-12	−1.89	58.71	BUB1B;BIRC5;NCAPD2;BUB1;AURKB;NDC80;SPC25
GO Celluar Component	condensed nuclear chromosome kinetochore (GO:0000778)	3.43E-14	3.41E-12	−1.71	53.02	BUB1B;BIRC5;BUB1;AURKB;NDC80;SPC25;AURKA
GO Celluar Component	polar microtubule (GO:0005827)	4.38E-13	3.26E-11	−2.13	60.63	TPX2;CCNB1;SPAG5;PRC1;KIF4A;CDK1;CDC6;KIF11;AURKB;AURKA
GO Celluar Component	mitotic spindle (GO:0072686)	5.47E-13	3.26E-11	−2.24	63.12	TPX2;SPAG5;PRC1;CDK1;TTK;KIF20A;KIF11;AURKB;AURKA;MAD2L1
GO Celluar Component	mitotic spindle midzone (GO:1990023)	8.40E-13	4.17E-11	−1.99	55.37	TPX2;SPAG5;CDK1;BUB1B;CDCA8;CDC6;AURKB;AURKA;MAD2L1
GO Celluar Component	mitotic spindle microtubule (GO:1990498)	2.46E-12	1.05E-10	−2.07	55.43	TPX2;SPAG5;PRC1;KIF4A;CDK1;KIF11;AURKB;AURKA;MAD2L1
GO Celluar Component	kinetochore microtubule (GO:0005828)	1.92E-11	7.15E-10	−1.7	41.94	SPAG5;PRC1;KIF4A;CDK1;KIF11;AURKB;AURKA
GO Biological process	spindle organization (GO:0007051)	2.68E-11	3.42E-09	−2.63	63.98	RANBP1;SPAG5;TTK;KIF11;AURKB;AURKA
GO Biological process	sister chromatid cohesion (GO:0007062)	3.00E-11	3.42E-09	−2.74	66.3	BUB1B;CDCA8;BIRC5;BUB3;BUB1;AURKB;NDC80;SPC25;MAD2L1
GO Celluar Component	spindle microtubule (GO:0005876)	4.08E-11	1.35E-09	−2.24	53.52	TPX2;SPAG5;PRC1;KIF4A;CDK1;TTK;KIF20A;KIF11;AURKB;AURKA
GO Celluar Component	spindle pole (GO:0000922)	5.66E-11	1.69E-09	−2.15	50.77	TPX2;CCNB1;PRC1;TTK;CDC6;KIF20A;KIF11;AURKB;AURKA
GO Celluar Component	spindle pole centrosome (GO:0031616)	1.05E-10	2.86E-09	−3.37	77.48	RANBP1;PCNA;CDC6;KIF11;AURKB;NDC80;AURKA;KIF15;TPX2;CCNB2;CCNB1;CHEK1;CDK1;DLGAP5
GO Biological process	anaphase-promoting complex-dependent catabolic process (GO:0031145)	2.18E-10	1.66E-08	−2.6	57.76	CCNB1;PTTG1;CDK1;BUB1B;BUB3;AURKB;AURKA;MAD2L1
GO Biological process	mitotic cell cycle (GO:0000278)	3.93E-10	2.24E-08	−2.78	60.2	TPX2;PBK;BUB1B;CDC6;KIF11;NDC80;AURKA;KIF15
GO Biological process	mitotic spindle organization (GO:0007052)	7.06E-10	3.22E-08	−2.88	60.61	CCNB1;TTK;KIF11;NDC80;SPC25;AURKA
GO Celluar Component	meiotic spindle (GO:0072687)	7.13E-10	1.77E-08	−1.86	39.08	TPX2;PRC1;TTK;KIF20A;KIF11;AURKB;AURKA
GO Celluar Component	mitotic spindle pole (GO:0097431)	3.29E-09	7.55E-08	−2.14	41.73	TPX2;CCNB1;SPAG5;CDK1;CDC6;KIF11;AURKA;MAD2L1
GO Biological process	protein ubiquitination involved in ubiquitin-dependent protein catabolic process (GO:0042787)	6.50E-09	2.47E-07	−2.97	56.06	CCNB1;PTTG1;CDK1;BUB1B;BUB3;AURKB;AURKA;MAD2L1
GO Celluar Component	condensed nuclear chromosome outer kinetochore (GO:0000942)	7.50E-09	1.60E-07	0.77	−14.35	CCNB1;BUB1B;BUB1;NDC80
GO Biological process	G2/M transition of mitotic cell cycle (GO:0000086)	8.39E-09	2.73E-07	−2.75	51.19	TPX2;CCNB2;CCNB1;MELK;CDK1;FOXM1;CDC25C;AURKA
GO Celluar Component	meiotic spindle midzone (GO:1990385)	1.36E-08	2.70E-07	−1.25	22.56	BUB1B;CDCA8;CDC6;AURKB;AURKA
GO Celluar Component	condensed chromosome, centromeric region (GO:0000779)	1.85E-08	3.45E-07	−1.61	28.65	CDCA8;BIRC5;OIP5;NCAPD2;AURKB;NDC80
GO Biological process	DNA replication (GO:0006260)	5.79E-08	0.000001651	−2.75	45.81	FEN1;CHEK1;CDK1;MCM10;DNA2;CDC6;CDC25C
GO Biological process	protein sumoylation (GO:0016925)	9.54E-08	0.000002418	−2.62	42.29	PCNA;TRIM28;CDCA8;BIRC5;SAE1;AURKB
GO Celluar Component	spindle (GO:0005819)	1.19E-07	0.000002088	−2.26	35.97	TPX2;CCNB2;PRC1;TTK;KIF20A;KIF11;AURKB;AURKA
GO Molecular Function	protein kinase binding (GO:0019901)	4.76E-07	0.00004189	−5.54	80.67	TPX2;CCNB1;PRC1;KIF20A;KIF11;FOXM1;CDC25C;TRIM22;AURKA
GO Biological process	chromosome segregation (GO:0007059)	5.15E-07	0.00001173	−2.27	32.94	SPAG5;OIP5;KIF11;NDC80;SPC25
GO Celluar Component	centriolar satellite (GO:0034451)	9.11E-07	0.00001507	−3.18	44.17	CCNB2;RANBP1;CCNB1;PCNA;SPAG5;CHEK1;CDK1;NDC80;AURKA;KIF15
GO Biological process	DNA damage response, signal transduction by p53 class mediator resulting in cell cycle arrest (GO:0006977)	0.000001877	0.00003609	−2.51	33.14	CCNB1;PCNA;CDK1;CDC25C;AURKA
GO Biological process	protein localization to kinetochore (GO:0034501)	0.0000019	0.00003609	−0.67	8.8	CDK1;BUB1B;AURKB
GO Celluar Component	centrosome (GO:0005813)	0.000002228	0.00003494	−3.18	41.37	CCNB2;RANBP1;CCNB1;PCNA;CHEK1;CDK1;DLGAP5;NDC80;AURKA;KIF15
GO Celluar Component	condensed nuclear chromosome, centromeric region (GO:0000780)	2.78E-06	4.14E-05	−1.37	17.58	CHEK1;NCAPD2;AURKB;AURKA
GO Biological process	attachment of spindle microtubules to kinetochore (GO:0008608)	0.000002843	0.00004986	−1.09	13.89	BUB3;AURKB;NDC80
GO Biological process	negative regulation of ubiquitin-protein ligase activity involved in mitotic cell cycle (GO:0051436)	0.000003956	0.00006443	−2.34	29.13	CCNB1;CDK1;BUB1B;BUB3;MAD2L1
GO Celluar Component	chromosome, centromeric outer repeat region (GO:0034507)	5.24E-06	7.10E-05	−1.08	13.07	CDCA8;BIRC5;OIP5;NDC80
GO Celluar Component	chromosome, centromeric region (GO:0000775)	0.000005243	0.00007102	−1.04	12.62	CDCA8;BIRC5;OIP5;NDC80
GO Biological process	positive regulation of ubiquitin-protein ligase activity involved in regulation of mitotic cell cycle transition (GO:0051437)	0.000005517	0.00008385	−2.29	27.77	CCNB1;CDK1;BUB1B;BUB3;MAD2L1
GO Celluar Component	chromosome, centromeric core domain (GO:0034506)	0.000005884	0.00007623	−1.13	13.61	CDCA8;BIRC5;OIP5;NDC80
GO Celluar Component	centrosomal corona (GO:0031592)	0.000006456	0.00008016	−3.03	36.22	CCNB2;RANBP1;CCNB1;PCNA;CHEK1;CDK1;NDC80;AURKA;KIF15
GO Celluar Component	pericentriolar material (GO:0000242)	0.000007133	0.00008502	−3.06	36.22	CCNB2;RANBP1;CCNB1;PCNA;CHEK1;CDK1;NDC80;AURKA;KIF15

**Note:** *GO* Gene Ontology

**Table 3:** KEGG enrichment analysis for genes in pink and blue modules

Series	Name	P-value	Adjusted p-value	Z-score	Combined score	GENE
KEGG	Cell cycle	1.63E-16	1.01E-14	−5.14	186.92	PCNA;BUB1B;TTK;CDC6;CDC25C;CCNB2;CCNB1;PTTG1;CHEK1;CDK1;BUB3;BUB1;MAD2L1
KEGG	Oocyte meiosis	7.88E-09	2.44E-07	−27.81	519.01	CCNB2;CCNB1;PTTG1;CDK1;CDC25C;BUB1;AURKA;MAD2L1
KEGG	Progesterone-mediated oocyte maturation	3.60E-08	6.41E-07	−16.58	284.12	CCNB2;CCNB1;CDK1;CDC25C;BUB1;AURKA;MAD2L1
KEGG	Human T-cell leukemia virus 1 infection	4.14E-08	6.41E-07	−20.7	351.91	CCNB2;RANBP1;HLA-DMA;PTTG1;IL1R1;CHEK1;BUB1B;BUB3;MAD2L1
KEGG	p53 signaling pathway	0.000094	0.001166	−7.32	67.86	CCNB2;CCNB1;CHEK1;CDK1
KEGG	Cellular senescence	0.0001856	0.001918	−3.15	27.08	CCNB2;CCNB1;CHEK1;CDK1;FOXM1
KEGG	DNA replication	0.0002267	0.002008	−48.57	407.62	FEN1;PCNA;DNA2
KEGG	Human immunodeficiency virus 1 infection	0.0006749	0.005231	−11.45	83.6	CCNB2;CCNB1;CHEK1;CDK1;CDC25C
KEGG	Base excision repair	0.005301	0.03652	−41.27	216.26	FEN1;PCNA

**Note:** *KEGG* Kyoto Encyclopedia of genes and Genomes

**Table 4:** GO functional annotation for genes green modules

Series	Name	P-value	Adjusted p-value	Z-score	Combined score	GENE
GO Biological process	excitatory postsynaptic potential (GO:0060079)	0.0004911	0.08474	−2.92	22.27	MAPK8IP2;GRIN1
GO Biological process	regulation of NMDA receptor activity (GO:2000310)	0.0007466	0.08474	−3.46	24.95	DLG4;MAPK8IP2
GO Celluar Component	excitatory synapse (GO:0060076)	0.0008938	0.03972	−2.75	19.28	DLG4;GRIN1
GO Celluar Component	postsynaptic density (GO:0014069)	0.001281	0.03972	−3.05	20.29	DLG4;MAPK8IP2;GRIN1
GO Biological process	calcium ion homeostasis (GO:0055074)	0.001819	0.1033	−2.92	18.41	TRPV6;GRIN1
GO Biological process	positive regulation of excitatory postsynaptic potential (GO:2000463)	0.001819	0.1033	−2.31	14.54	DLG4;GRIN1
GO Biological process	stress-activated MAPK cascade (GO:0051403)	0.003053	0.1372	−2.45	14.17	TAOK2;MAP 2 K7
GO Biological process	phosphatidylinositol 3-kinase signaling (GO:0014065)	0.004254	0.1372	−2.45	13.38	LTK;PIK3CD
GO Biological process	signal transduction (GO:0007165)	0.004805	0.1372	−6.49	34.63	PSD;CACNA1I;LTK;EPO;DLG4;PIK3CD;IGFALS;RASGRP2;MAP 2 K7
GO Celluar Component	postsynaptic membrane (GO:0045211)	0.006795	0.1289	−2.47	12.32	DLG4;GRIN1
GO Molecular Function	triglyceride lipase activity (GO:0004806)	0.007203	0.2384	−1.95	9.61	CES1P1;PNPLA2

**Note:** *GO* Gene Ontology

**Table 5:** KEGG enrichment analysis for genes green modules

Series	Name	P-value	Adjusted p-value	Z-score	Combined score	Genes
KEGG	Ras signaling pathway	0.001795	0.2316	−12.94	81.81	PIK3CD;FOXO4;RASGRP2;RIN1;GRIN1
KEGG	MAPK signaling pathway	0.005023	0.2351	−12.02	63.64	CACNA1I;TAOK2;RASGRP2;MAP 2 K7;MAPK8IP2
KEGG	Calcium signaling pathway	0.005468	0.2351	−1.88	9.78	CACNA1I;LTB4R2;PTAFR;GRIN1

**Note:** *KEGG* Kyoto Encyclopedia of genes and Genomes

### Hub gene evaluation

**Fig. 8:**
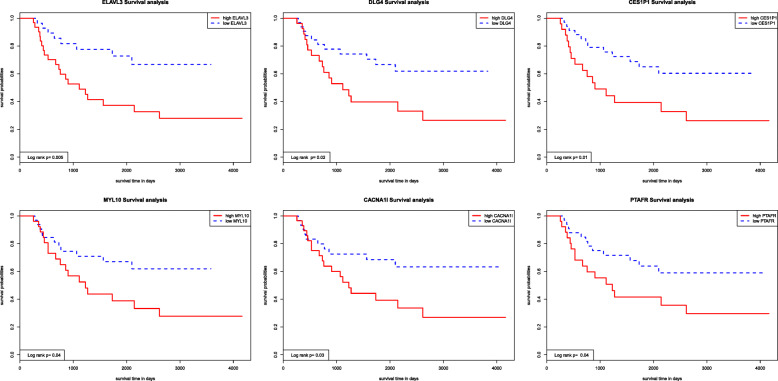
Overall survival analyses on hub genes. Note: The red lines represent high expression of hub genes, while blue lines represent low expression of hub genes

**Fig. 9:**
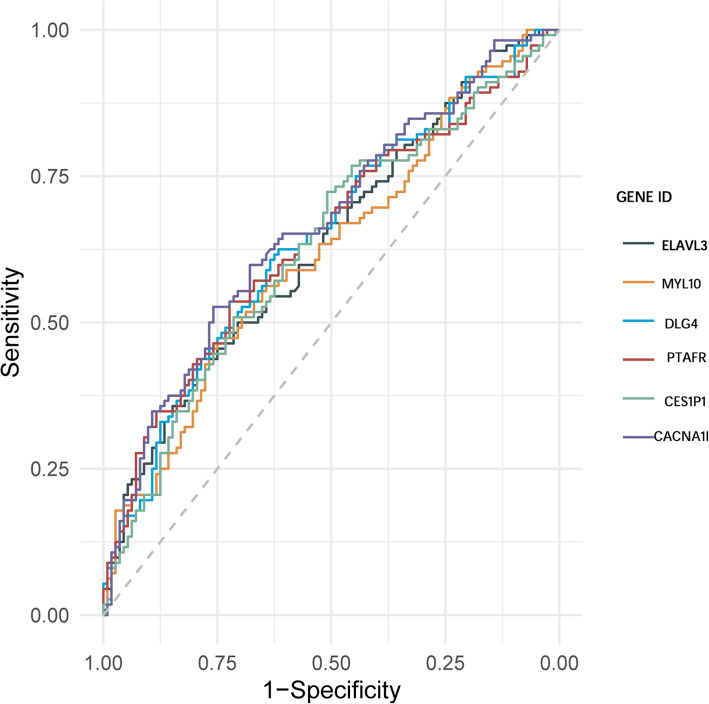
ROC curve value of 6 hub genes. Note: Different colored curves represent different genes

All patients were divided into two groups according to the median expression value of each hub genes in the three modules. The corresponding Kaplan-Meier estimate value was calculated, and the Kaplan-Meier survival curve was plotted. A log-rank test showed that the difference in survival time between the two groups corresponding to 6 hub genes (*P* < 0.05) in the green module was statistically significant (Fig. 8). The reason for this result may be that among three modules, the green module is most relevant to the overall survival time (Fig. 3). The image of the high-expression group of 6 hub genes was steeper than that of the low-expression group, indicating that the high expression of these hub genes was closely related to the poor prognosis of patients. In addition, we drew ROC curves for the six hub genes, as shown in Fig. 9 and Table 6, and the six hub genes had moderate diagnostic value.

**Table 6:** ROC curve value of 6 hub genes

Gene symbol	AUC(95%CI)
ELAVL3	0.6344 (0.5621,0.7067)
MYL10	0.6193 (0.5431,0.6926)
DLG4	0.6451 (0.5731,0.7171)
PTAFR	0.6435 (0.5711,0.7159)
CES1P1	0.629 (0.5559,0.7021)
CACNA1I	0.6659 (0.5952,0.7365)

## Discussion

WT is one of the most common tumors in children, and although modern multimodality therapy can improve the survival rate of WT patients, not all patients are spared. At present, the stage of WT can refer to the degree of resection, perioperative rupture of the tumor capsule, lymphatic diffusion and distant metastasis [1]. The chromosome 11p contains two biomarkers of concern: 11p13 (WT1) and 11p15.5 (WT2). WT1 mutation occurs in 15% of sporadic patients, loss of heterozygosity (LOH) at chromosome 11p15.5 accounts for 70% of WT patients [17]. Loss of the entire longarm of chromosome 11 was associated with higher rates of relapse and death [18]. Chromosomes 1p and 16q are also regions of concern for genetic changes. Multiple studies have suggested that LOH at 1p and/or 16q associates with relapse and over all poor prognosis [19, 20]. So far, combined LOH at 1p/16q is the only molecular marker used for risk stratification. Although LOH at 1p and 16q was sensitive in predicting recurrence, this combination was present in only 9.4% of recurrent tumors [1]. Therefore, there is still some room to learn about WT.

In this study, a dataset including 59 WT samples were used to construct the co-expression network, and 13 modules were identified, among which the green module was most related to stage, while the blue module and pink module were tightly related to event-free (recurrence) survival time. After further analysis, we identified 11 hub genes in the pink module, 15 hub genes in the green module, and 18 hub genes in the blue module for a total of 44 hub genes. It is worth mentioning that differential expression of 44 hub gene in other independent data sets is statistically significant. Gene enrichment pathways in the blue and pink modules were mainly focused on those related to sister chromatid cohesion, cell division and proliferation. Sister chromatid cohesion is an important pathway mediated by cohesive proteins to ensure normal chromosome segregation in cells [21]. We have not retrieved studies on the mechanism of sister chromatid cohesion and WT, but sister chromatid cohesion has been reported to be frequently amplified in liver cancer cells, which might a driver gene promoting the proliferation of liver cancer cells and related to poor prognosis of liver cancer. Survival rates in breast cancer patients were associated with sister chromatid cohesion [22]. In addition, sister chromatid cohesion has been implicated in studies of pancreatic, bladder, and colorectal cancers. One report suggests that identifying cancer cells with sister chromatid cohesion mutations may be a new therapeutic opportunity [23]. Genes in green module enrichment into the Ras signaling pathway that play an important role in tumor progression through proliferation, survival, invasion and immune escape [24]. The Ras signaling pathway is mutated and highly activated in thyroid cancer, melanoma and many other cancers. The gene in the green module has also been enriched to excitatory postsynaptic potential and excitatory synapse, and relatively little research has been performed on these pathways and cancers.

CDK1 is a prominent member of the cell cycle and is the most connected gene in the PPI network of blue and pink modules. CDK1 is a member of cyclin-dependent protein kinases (CDKs), which was mentioned in the pathogenesis and recurrence mechanism of various malignant tumors. CDKs and the cell cycle protein, as important proteins, are essential to the control and express the cycle [25]. CDK1 is mainly responsible for the G1/S and G2/M cell-cycle transitions. Many studies have shown that increased expression of CDKs or endogenous CDK regulator/inhibitor levels drop in various cancers, hematology tumor and sarcomas are visible, and because CDKs are natural targets for the treatment of cancer, many studies have shown that CDK inhibitors (e.g., AT7519) can inhibit cancer progression [26]. In the case of CDK1, its down-regulation may induce mitotic mutations leading to apoptosis of WT cells [27]. In addition, CDK1 is associated with poor prognosis in human pharyngeal squamous cell carcinoma [28], prostate cancer [29], ovarian cancer [30], oral squamous cell carcinoma [31], pancreatic ductal adenocarcinoma [32] and other cancers. The recognized cancer genes CCNB1 and CCNB2 were also identified as WT-related genes. The hub gene in the pink module, TRIM28, is one of the susceptibility genes of WT. TRIM28 cells showed positive immunohistochemical staining in WT cells but negative staining in other tissues and WT epithelial components [33]. This gene was identified as the new susceptibility gene of wilms tumor in a study based on 890 wilms tumor patients with lymphocyte DNA exome sequencing [34], acting as a tumor suppressor gene by LOH [35]. Considering the above reasons, some scholars suggested that patients with epithelial wilms tumor should undergo TRIM28 gene detection [34].

Although there has been less research on the hub genes in the blue and pink modules of WT, there has been even less research on the hub genes in the green modules and WT compared to the blue and pink modules. Research on these hub genes is imperative to fully elucidate how alterations in cell differentiation relate to WT. The 6 hub genes of the green module (ELAVL3, DLG4, CES1P1, MYL10, CACNA1 and PTAFR) that were shown to have statistically significant differences in survival analysis and moderate diagnostic value in ROC curve were the focus of our attention. PTAFR have been reported as biomarkers for breast cancer [36]. PTAFR is a platelet activating factor receptor associated with many characteristic and inflammatory diseases, but it has been less frequently reported in cancer [37, 38]. CES1P1 are associated with the progression of gastric cancer. DLG4 is associated with poor prognosis for prostate cancer and colorectal cancer. Compared with the genes in the blue and pink modules, the genes in the green module have been poorly studied, not only in relation to WT but also to other cancers. Therefore, further investigating the genes in the green module may be the direction of our future research.

Compared with a study using the WGCNA method to identify hub genes associated with WT prognosis [39], our study samples were all from NCI. Of the 44 hub genes we identified, CDK1 and CDCA8 are also hub genes in that study, which also verified these two genes. Although there are similarities, we found many new genes that are closely related to WT pathological staging and poor prognosis. Many of these new genes are associated with the relapse of WT, which is currently one of the leading causes of death in WT patients. To the best of our knowledge, our study is the first to use the WGCNA method to identify genes associated with WT recurrence. In a study on WT using the MTT assay and clonal survival assay, mir-1180 was up-regulated in WT and may be a therapeutic target for WT in the future [40]. Another study showed that the hypomethylation level of long interspersed element-1 in WT could be used as a marker of recurrence after chemotherapy [41]. Glypican-3, which is specifically expressed in cancers including WT, is being considered as a biomarker for predicting tumor recurrence [42]. In a study of constructing a DEGs-Transcription Factors-miRNA network to explore WT-related biomolecular markers, and TFs and miRNA were mainly studied, but this study focused on hub genes [43]. Our study identified new biomarkers associated with WT recurrence, which may be a new research direction in the future. However, our study has some limitations: (1) Due to the small number of samples and possible bias, we need to conduct considerable research in the future to verify our results. (2) The original NCI data did not provide the data of progression-free survival, which prevented this study from evaluating this important clinical outcome.

## Conclusions

In summary, with the help of WGCNA, PPI network model construction, GO analysis, KEGG analysis and survival analysis, we identify hub genes are closely to recurrence and staging of WT, and 6 of these hub genes were strongly associated with overall survival. Our study may be of great significance for potential susceptibility gene of WT, which may improve the patient’s recurrence and prognosis by adjusting the clinical treatment regimen.

## Supplementary Information

**Additional file 1.** Analysis of network topology for various soft- thresholding powers. Note: The left panel showed the scale-free fit index, signed R^2 (y-axis) and the soft threshold power (x-axis).

**Additional file 2.** Dendrogram of all differentially expressed genes clustered based on a dissimilarity measure (1-TOM).

**Additional file 3.** Scatter diagram for module membership vs. gene significance of stage in green module.

**Additional file 4.** Scatter diagram for module membership vs. gene significance for event free survival time in blue module.

**Additional file 5.** Scatter diagram for module membership vs. gene significance for event free survival time in pink module.

## Data Availability

Raw gene expression profiles were downloaded from NCI (https://ocg.cancer.gov/programs/target/data-matrix). All tissue samples are from the platform named GPL96 [HG-U133A] Affymetrix Human Genome U133A Array.

## Abbreviations

ccRCC: Clear cell renal cell carcinoma
CDKs: Cyclin-dependent protein kinases
GO: Gene ontology
GS: Gene significance
HCC: Hepatocellular carcinoma
KEGG: Kyoto Encyclopedia of Genes and Genomes
ME: Modules eigengene
MS: Module significance
NCI: National Cancer Institute
RMA: Robust multi-array averaging
TOM: Topological overlap matrix
WT: Wilms tumor
WGCNA: Weighted gene coexpression network analysis

## References

[1] Phelps HM, Kaviany S, Borinstein SC, Lovvorn HN (2018). Biological drivers of Wilms tumor prognosis and treatment. Children (Basel).

[2] Treger TD, Chowdhury T, Pritchard-Jones K, Behjati S (2019). The genetic changes of Wilms tumour. Nat Rev Nephrol.

[3] Dix DB, Fernandez CV, Chi YY, Mullen EA, Geller JI, Gratias EJ (2019). Augmentation of therapy for combined loss of Heterozygosity 1p and 16q in favorable histology Wilms tumor: a Children's oncology group AREN0532 and AREN0533 study report. J Clin Oncol.

[4] Fernandez CV, Perlman EJ, Mullen EA, Chi YY, Hamilton TE, Gow KW (2017). Clinical outcome and biological predictors of relapse after nephrectomy only for very low-risk Wilms tumor: a report from Children's oncology group AREN0532. Ann Surg.

[5] Holl EK, Routh JC, Johnston AW, Frazier V, Rice HE, Tracy ET (2019). Immune expression in children with Wilms tumor: a pilot study. J Pediatr Urol.

[6] Spreafico F, Pritchard Jones K, Malogolowkin MH, Bergeron C, Hale J, de Kraker J (2009). Treatment of relapsed Wilms tumors: lessons learned. Expert Rev Anticancer Ther.

[7] Malogolowkin M, Cotton CA, Green DM, Breslow NE, Perlman E, Miser J (2008). Treatment of Wilms tumor relapsing after initial treatment with vincristine, actinomycin D, and doxorubicin. A report from the National Wilms Tumor Study Group. Pediatr Blood Cancer.

[8] Langfelder P, Horvath S (2008). WGCNA: an R package for weighted correlation network analysis. BMC Bioinformatics.

[9] Irizarry RA, Hobbs B, Collin F, Beazer-Barclay YD, Antonellis KJ, Scherf U (2003). Exploration, normalization, and summaries of high density oligonucleotide array probe level data. Biostatistics..

[10] Zhao W, Langfelder P, Fuller T, Dong J, Li A, Hovarth S (2010). Weighted gene coexpression network analysis: state of the art. J Biopharm Stat.

[11] Zhang B, Horvath S (2005). A general framework for weighted gene co-expression network analysis. Stat Appl Genet Mol Biol.

[12] Langfelder P, Luo R, Oldham MC, Horvath S (2011). Is my network module preserved and reproducible?. PLoS Comput Biol.

[13] Guo SM, Wang JX, Li J, Xu FY, Wei Q, Wang HM (2018). Identification of gene expression profiles and key genes in subchondral bone of osteoarthritis using weighted gene coexpression network analysis. J Cell Biochem.

[14] Jeong H, Mason SP, Barabasi AL, Oltvai ZN (2001). Lethality and centrality in protein networks. Nature..

[15] Kuleshov MV, Jones MR, Rouillard AD, Fernandez NF, Duan Q, Wang Z (2016). Enrichr: a comprehensive gene set enrichment analysis web server 2016 update. Nucleic Acids Res.

[16] Goel MK, Khanna P, Kishore J (2010). Understanding survival analysis: Kaplan-Meier estimate. Int J Ayurveda Res.

[17] Brok J, Treger TD, Gooskens SL, van den Heuvel-Eibrink MM, Pritchard-Jones K (2016). Biology and treatment of renal tumours in childhood. Eur J Cancer.

[18] Wittmann S, Zirn B, Alkassar M, Ambros P, Graf N, Gessler M (2007). Loss of 11q and 16q in Wilms tumors is associated with anaplasia, tumor recurrence, and poor prognosis. Genes Chromosomes Cancer.

[19] Grundy RG, Pritchard J, Scambler P, Cowell JK (1998). Loss of heterozygosity on chromosome 16 in sporadic Wilms' tumour. Br J Cancer.

[20] Spreafico F, Gamba B, Mariani L, Collini P, D'Angelo P, Pession A (2013). Loss of heterozygosity analysis at different chromosome regions in Wilms tumor confirms 1p allelic loss as a marker of worse prognosis: a study from the Italian Association of Pediatric Hematology and Oncology. J Urol.

[21] Kim JS, He X, Liu J, Duan Z, Kim T, Gerard J (2019). Systematic proteomics of endogenous human cohesin reveals an interaction with diverse splicing factors and RNA binding proteins required for mitotic progression. J Biol Chem.

[22] Repo H, Loyttyniemi E, Nykanen M, Lintunen M, Karra H, Pitkanen R (2016). The expression of Cohesin subunit SA2 predicts breast cancer survival. Appl Immunohistochem Mol Morphol.

[23] Mintzas K, Heuser M. Emerging strategies to target the dysfunctional cohesin complex in cancer. Expert Opin Ther Targets. 2019;23(6):525–37. 10.1080/14728222.2019.1609943 31020869

[24] Mizukami T, Izawa N, Nakajima TE, Sunakawa Y (2019). Targeting EGFR and RAS/RAF signaling in the treatment of metastatic colorectal cancer: from current treatment strategies to future perspectives. Drugs..

[25] Squires MS, Feltell RE, Wallis NG, Lewis EJ, Smith DM, Cross DM (2009). Biological characterization of AT7519, a small-molecule inhibitor of cyclin-dependent kinases, in human tumor cell lines. Mol Cancer Ther.

[26] Xi C, Wang L, Yu J, Ye H, Cao L, Gong Z (2019). Inhibition of cyclin-dependent kinases by AT7519 is effective to overcome chemoresistance in colon and cervical cancer. Biochem Biophys Res Commun.

[27] Du M, Qiu Q, Gruslin A, Gordon J, He M, Chan CC (2013). SB225002 promotes mitotic catastrophe in chemo-sensitive and -resistant ovarian cancer cells independent of p53 status in vitro. PLoS One.

[28] Hung KC, Wang SG, Lin ML, Chen SS. Citrate-induced p85alpha(−)PTEN complex formation causes G2/M phase arrest in human pharyngeal squamous carcinoma cell lines. Int J Mol Sci. 2019;20(9). 10.3390/ijms20092105. 10.3390/ijms20092105 PMC6539620 31035650

[29] Makarevic J, Rutz J, Juengel E, Maxeiner S, Tsaur I, Chun FK, et al. Influence of the HDAC inhibitor Valproic acid on the growth and proliferation of Temsirolimus-resistant prostate cancer cells in vitro. Cancers (Basel). 2019;11(4):566. 10.3390/cancers11040566 PMC6520872 31010254

[30] Liu J, Li S, Liang J, Jiang Y, Wan Y, Zhou S (2019). ITLNI identified by comprehensive bioinformatic analysis as a hub candidate biological target in human epithelial ovarian cancer. Cancer Manag Res.

[31] Mohanta S, Sekhar Khora S, Suresh A (2019). Cancer stem cell based molecular predictors of tumor recurrence in Oral squamous cell carcinoma. Arch Oral Biol.

[32] Piao J, Zhu L, Sun J, Li N, Dong B, Yang Y (2019). High expression of CDK1 and BUB1 predicts poor prognosis of pancreatic ductal adenocarcinoma. Gene..

[33] Halliday BJ, Fukuzawa R, Markie DM, Grundy RG, Ludgate JL, Black MA (2018). Germline mutations and somatic inactivation of TRIM28 in Wilms tumour. PLoS Genet.

[34] Mahamdallie S, Yost S, Poyastro-Pearson E, Holt E, Zachariou A, Seal S (2019). Identification of new Wilms tumour predisposition genes: an exome sequencing study. Lancet Child Adolesc Health.

[35] Diets IJ, Hoyer J, Ekici AB, Popp B, Hoogerbrugge N, van Reijmersdal SV (2019). TRIM28 haploinsufficiency predisposes to Wilms tumor. Int J Cancer.

[36] Hou T, Lou Y, Li S, Zhao C, Ji Y, Wang D (2018). Kadsurenone is a useful and promising treatment strategy for breast cancer bone metastases by blocking the PAF/PTAFR signaling pathway. Oncol Lett.

[37] Chase PB, Yang JM, Thompson FH, Halonen M, Regan JW (1996). Regional mapping of the human platelet-activating factor receptor gene (PTAFR) to 1p35-->p34.3 by fluorescence in situ hybridization. Cytogenet Cell Genet.

[38] Liu L, Chen F, Xiu A, Du B, Ai H, Xie W (2018). Identification of key candidate genes and pathways in endometrial cancer by integrated Bioinformatical analysis. Asian Pac J Cancer Prev.

[39] Wang X, Song P, Huang C, Yuan N, Zhao X, Xu C (2019). Weighted gene coexpression network analysis for identifying hub genes in association with prognosis in Wilms tumor. Mol Med Rep.

[40] Jiang X, Li H (2018). MiR-1180-5p regulates apoptosis of Wilms' tumor by targeting p73. Onco Targets Ther.

[41] de Sa Pereira BM, Montalvao-de-Azevedo R, Faria PA, de Paula SN, Nicolau-Neto P, Maschietto M (2017). Association between long interspersed nuclear element-1 methylation levels and relapse in Wilms tumors. Clin Epigenetics.

[42] Shimizu Y, Suzuki T, Yoshikawa T, Endo I, Nakatsura T (2019). Next-generation cancer immunotherapy targeting Glypican-3. Front Oncol.

[43] Chen W, Zhuang J, Gong L, Dai Y, Diao H (2019). Investigating the dysfunctional pathogenesis of Wilms' tumor through a multidimensional integration strategy. Ann Transl Med.

