# Supplementary material for: Identification of the potential novel biomarkers as susceptibility gene for Wilms tumor
Source: BMC Cancer. 2021 Mar 25;21:316. doi: 10.1186/s12885-021-08034-w (PMC7992941; doi:10.1186/s12885-021-08034-w)
Supplement: Supplementary file 1 — Additional file 1. Analysis of network topology for various soft- thresholding powers. Note: The left panel showed the scale-free fit index, signed R^2 (y-axis) and the soft threshold power (x-axis). [file 12885_2021_8034_MOESM1_ESM.docx]

**Additional file 1** Analysis of network topology for various soft- thresholding powers.

**
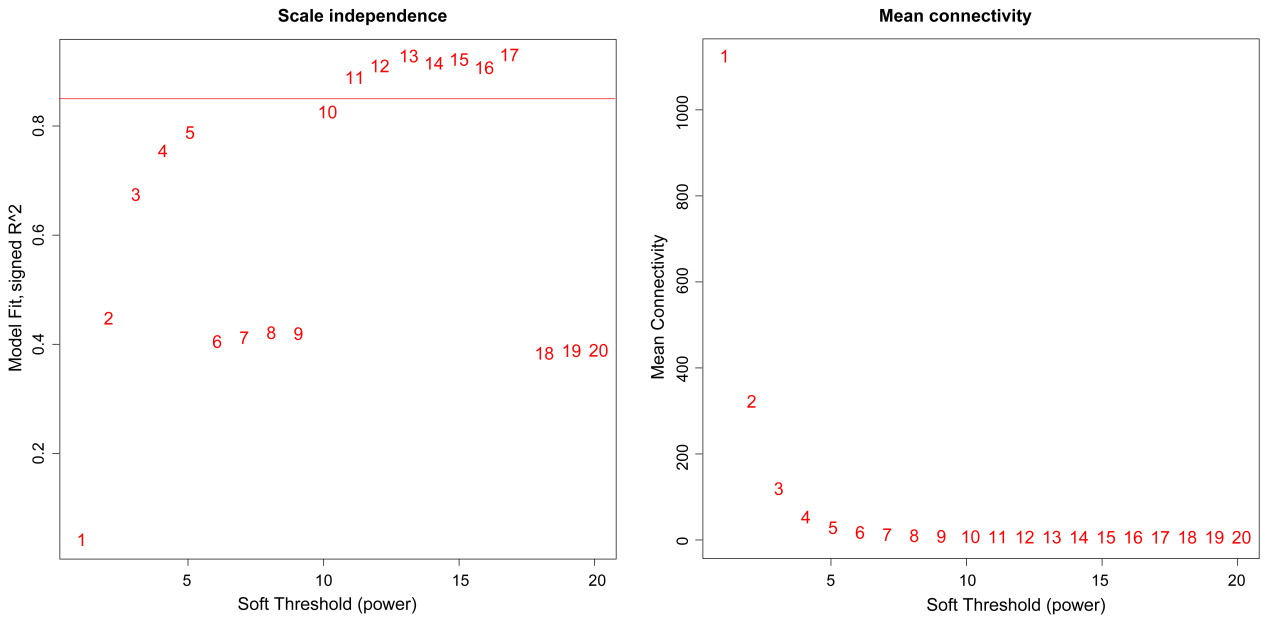
**

**Note:** The left panel showed the scale-free fit index, signed R^2 (y-axis) and the soft threshold power (x-axis).
