# Supplementary material for: Identification of the potential novel biomarkers as susceptibility gene for Wilms tumor
Source: BMC Cancer. 2021 Mar 25;21:316. doi: 10.1186/s12885-021-08034-w (PMC7992941; doi:10.1186/s12885-021-08034-w)

**Additional file 2** Dendrogram of all differentially expressed genes clustered based on a dissimilarity measure (1-TOM).


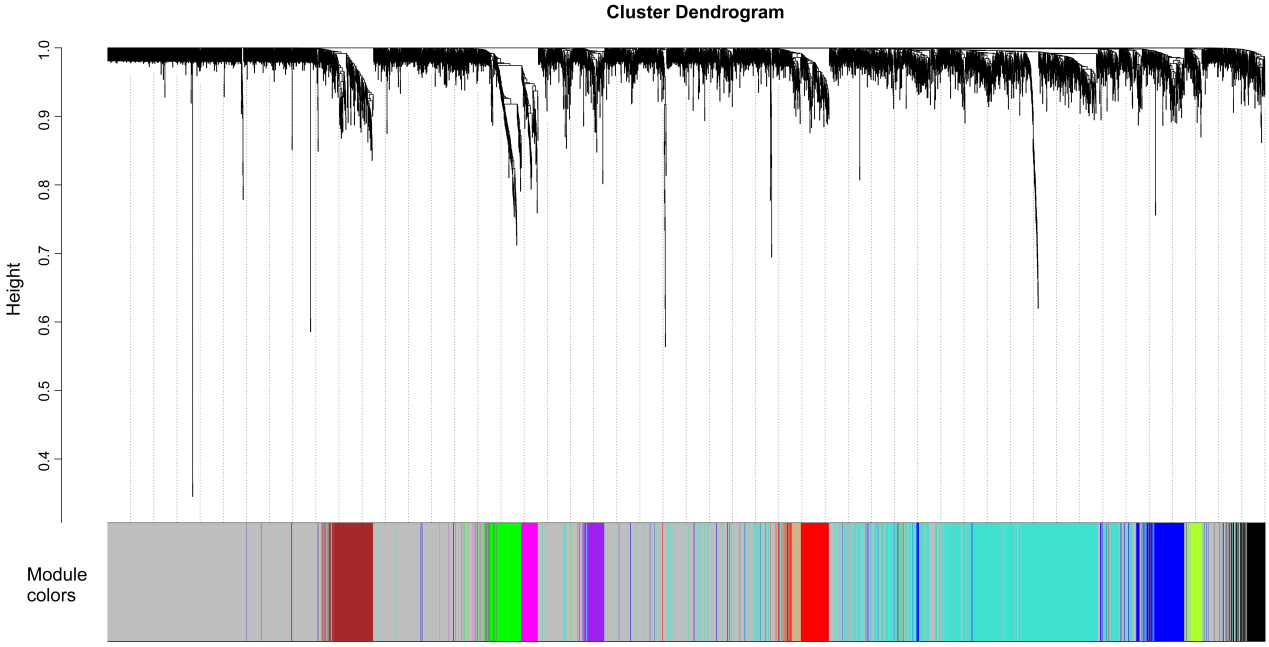

Supplement: Supplementary file 2 — Additional file 2. Dendrogram of all differentially expressed genes clustered based on a dissimilarity measure (1-TOM). [file 12885_2021_8034_MOESM2_ESM.docx]
