# Supplementary material for: Identification of the potential novel biomarkers as susceptibility gene for Wilms tumor
Source: BMC Cancer. 2021 Mar 25;21:316. doi: 10.1186/s12885-021-08034-w (PMC7992941; doi:10.1186/s12885-021-08034-w)

**Additional file 4** Scatter diagram for module membership vs. gene significance for event free survival time in blue module.


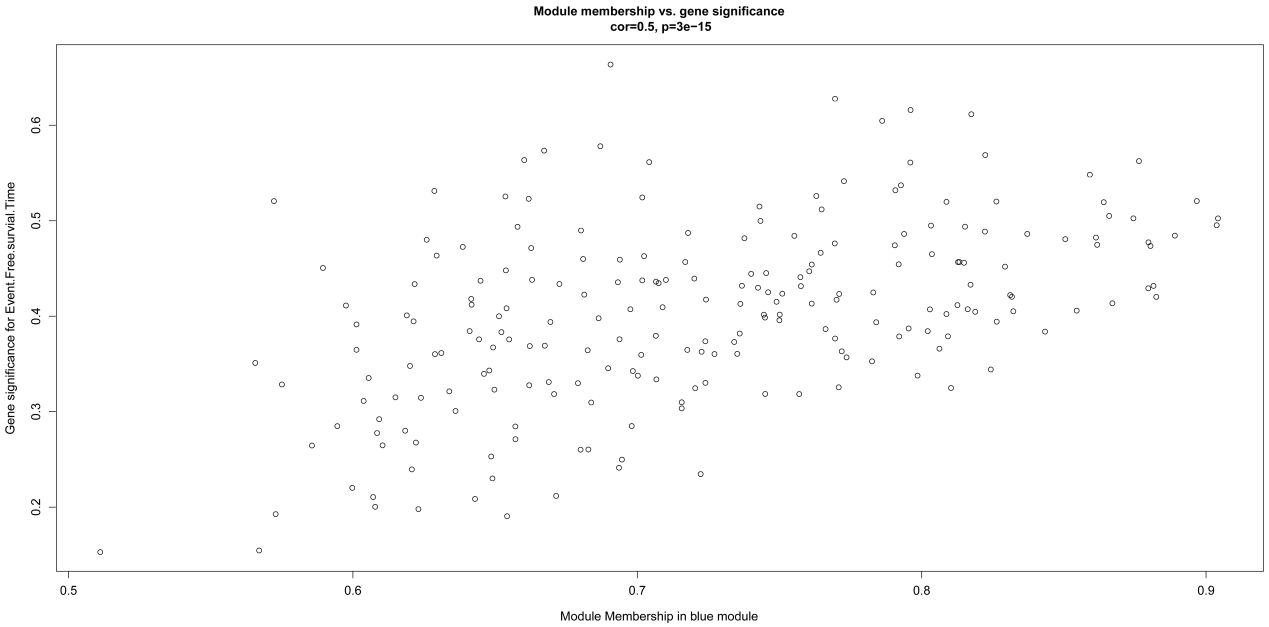

Supplement: Supplementary file 4 — Additional file 4. Scatter diagram for module membership vs. gene significance for event free survival time in blue module. [file 12885_2021_8034_MOESM4_ESM.docx]
